# Fabrication, Flow Control, and Applications of Microfluidic Paper-Based Analytical Devices

**DOI:** 10.3390/molecules24162869

**Published:** 2019-08-07

**Authors:** Hosub Lim, Ali Turab Jafry, Jinkee Lee

**Affiliations:** 1 School of Mechanical Engineering, Sungkyunkwan University, Suwon, Gyeonggi-do 16419, Korea; 2 Faculty of Mechanical Engineering, Ghulam Ishaq Khan Institute of Engineering Sciences and Technology, Topi 23640, Pakistan; 3 Institute of Quantum Biophysics, Sungkyunkwan University, Suwon, Gyeonggi-do 16419, Korea

**Keywords:** microfluidics, µPAD, 2D µPADs, 3D µPADs, fabrication, paper-based analytical device

## Abstract

Paper-based microfluidic devices have advanced significantly in recent years as they are affordable, automated with capillary action, portable, and biodegradable diagnostic platforms for a variety of health, environmental, and food quality applications. In terms of commercialization, however, paper-based microfluidics still have to overcome significant challenges to become an authentic point-of-care testing format with the advanced capabilities of analyte purification, multiplex analysis, quantification, and detection with high sensitivity and selectivity. Moreover, fluid flow manipulation for multistep integration, which involves valving and flow velocity control, is also a critical parameter to achieve high-performance devices. Considering these limitations, the aim of this review is to (i) comprehensively analyze the fabrication techniques of microfluidic paper-based analytical devices, (ii) provide a theoretical background and various methods for fluid flow manipulation, and (iii) highlight the recent detection techniques developed for various applications, including their advantages and disadvantages.

## 1. Introduction

Analytical testing using paper has a long history, such as the litmus test dating back to the early 18^th^ century [1]. Paper patterning with hydrophobic materials can be traced back to the early 19^th^ century where reaction zones were fabricated with paraffin [2,3]. However, it was only in 2007 [4] that the field of paper-based microfluidics emerged as a promising alternative to the conventional microfluidic devices for disease diagnostics, drug analysis, environmental monitoring, and food quality control, offering affordable, portable, and biodegradable analytical devices [5,6]. The inherent advantage in using paper lies in its capillary action, which results in an effortless fluid flow without the requirement for external pumping [7]. A paper channel circumvents the air bubble issue faced by conventional microfluidic channels and it can also serve as a pumping source for these channels [8,9,10,11,12].

Microfluidic paper-based analytical devices (μPADs) can analyze not only simple lateral flow tests but also perform complex analysis requiring multiple stage reactions using only small amounts of biochemical samples. The fabrication techniques, fluid manipulation, and detection capabilities for low concentrations of analytes have improved considerably in the past decade involving 1D, 2D, and 3D devices for a variety of applications [13].

**Figure 1:**
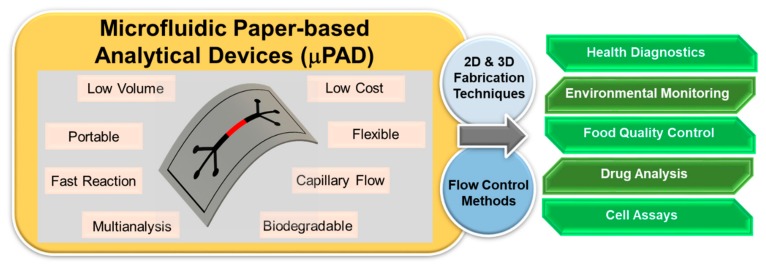
Schematic diagram of the advantages and potential applications of 2D & 3D microfluidic paper-based analytical devices.

This review focuses on the recent advancements in the areas of novel fabrication techniques, fluid handling, and detection methodologies for biomedical, environmental, and food quality control applications (Figure 1). The fabrication techniques are analyzed extensively in this article. Fluid handling is a crucial element in achieving an authentic point-of-care (POC) device; hence, it merits a critical review. The applications are linked to the various detection techniques of paper-based microfluidics and are presented in this paper along with their challenges and future directions. We have discussed the positive as well as negative aspects of the included literature. As each category is independently a vast field with several new developments constantly emerging, it is impossible to include all the literature in one review article. Hence, we have attempted to focus on the most prominent works.

## 2. Fabrication Techniques

Based on recent advancements in the field of paper-based microfluidics, the fabrication techniques can be broadly categorized into two types: (i) chemical patterning for creating barriers by blocking the pores inside the paper and (ii) physical patterning or cutting to form a defined channel shape. These techniques are summarized in Table 1.

### 2.1. Chemical Patterning

**Photolithography:** The first chip fabricated using photolithography was reported by Whiteside and his group in 2007 [4]. Their idea was to utilize a single platform to detect multiple assays as opposed to the conventional lateral flow paper strips. Moreover, photolithography was chosen for its convenience and the eventual plan was to move to other printing technologies as discussed in the subsequently listed fabrication techniques. The traditional photolithography process was conducted by soaking a chromatography paper in SU-8 photoresist and heating it to remove the cyclopentanone. It was then exposed to UV light through a photomask followed by a post-bake at 95 °C for crosslinking the exposed area of the photoresist. As the photoresist is imbedded within the paper at this stage, the cleaning process used propylene glycol monomethyl ether acetate and propanol followed by oxygen plasma to make it hydrophilic.

To create a rapid, convenient, and cost-effective process, Whiteside and co-workers further simplified their process to develop another method called fast lithographic activation of sheets (FLASH) [14]. FLASH replaced the conventional expensive photolithography equipment and clean room settings with a simple UV lamp and a hot plate. Another method used a hydrophobic surface created by thermally depositing TiO_2_ nanoparticles (NPs). A mask was then used to irradiate UV light onto the photocatalytic TiO_2_ NPs for generating hydrophilic channels [15].

A laser-based fabrication method was investigated by Sones et al. [16]. The light-sensitive polymer-soaked paper was patterned directly using a UV laser, achieving a significantly high resolution of 80 μm in the fluidic channel for paper-based microfluidic devices.

Recently, Rapp et al. demonstrated a photolithographic method for forming hydrophobic barriers using a mixture of silane and photosensitizer [17]. This method demonstrated a fast processing time and better flexibility owing to the process of locally modifying the wetting positions.

**Figure 2:**
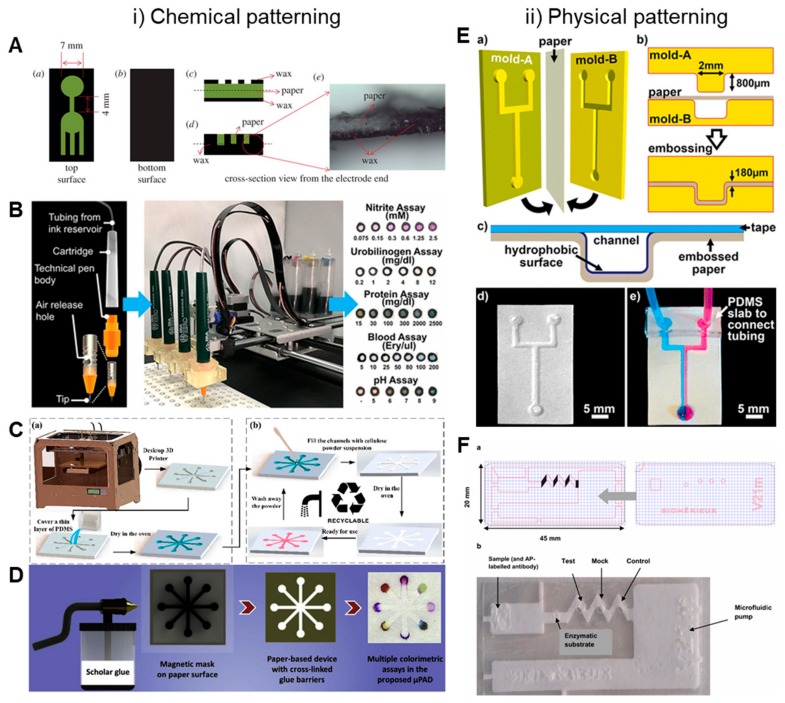
Recent 2D paper-based microfluidic devices. **i**) Chemical patterning: (**A**) double-sided wax printing (Suresh et al., reprinted with permission from ref [25]. Copyright 2018, the Royal Society), (**B**) continuous-ink, multiplexed pen-plotter (reprinted with permission from ref [33] Copyright 2017 American Chemical Society), (**C**) 3D printing (reproduced with permission from He et al., Micromachines; published by MDPI, 2016) [67], and (**D**) scholar glue spraying (reprinted from ref [70] with permission from Elsevier, Copyright 2017) methods to fabricate a liquid barrier. **ii**) Physical patterning: (**E**) embossing (reprinted with permission from ref [76]. Copyright 2014 American Chemical Society) and (**F**) laser-cutting (reprinted from ref [80] with permission from Springer, Copyright 2018) methods to fabricate liquid channels.

**Wax Printing:** The expensive nature of the photolithography process and its contamination within the hydrophilic channel resulted in it being less attractive for mass production. However, its principle of creating barriers within the porous paper can be replicated using other cost-effective materials. Hence, wax was the first of those materials to be tested within the paper. The wax can be patterned using either a pen or a wax printer [18,19,20,21,22,23]. It is then heated in an oven or a hot plate, which allows the wax to penetrate through the paper and create a hydrophobic barrier. Later, Kevin et al. developed an enclosing method to avoid contamination of the open wax-printed channels [24]. They printed both the top and bottom sides of the wax-patterned paper using a printing toner, which permitted the device to be easily handled and additionally prohibited the evaporation of the liquid reagents, which saved the solution and maintained the level of concentration (Figure 2A) [25].

Other similar methods used a metallic mask to transfer the pattern onto the paper surface using molten wax [26,27]. Songjaroen et al. used laser cutting for creating an iron mold and sandwiched the paper between the mask and a glass surface. The entire assembly was dipped in molten wax for a second and taken out. Once the paper cooled, the glass and mask were removed to attain a patterned μPAD.

**Plasma Treatment:** To make the paper hydrophobic, it was first immersed into alkyl ketene dimer (AKD) with heptane solution, in a previous study [28]. It was allowed to dry and then heated at 100 °C for 45 min for curing the AKD, resulting in an entirely hydrophobic paper. The hydrophilic regions were then defined by plasma treatment with the help of metallic masks with the desired channel layout. Furthermore, plasma treatment was used to control the wettability of patterned paper, to create paper-based circuits [29]. In another method, fluorocarbon plasma polymerization was alternatively used for making the hydrophobic channel boundaries [30]. Positive and negative metal masks were used for better confinement of the liquid by the hydrophobic walls surrounding it from three sides instead of two. The advantages of this treatment are the simplicity of fabrication as well as the cost effectiveness of the reagents. However, the metal masks can be expensive; the plasma treatment also requires vacuum and expensive instrumentation. Circumventing these limitations, Kao et al. developed a portable, inexpensive, battery-operated microplasma generator device for fabricating μPADs [31].

**Plotting:** Penplotting uses an x-y plotter or a template to fabricate μPADs (Figure 2B) [32,33,34]. Hydrophobic ink is used, such as polydimethylsiloxane (PDMS) dissolved in hexane, to print on the surface of the paper. The achieved barrier thickness in this case is 1 mm. The advantages of this technique are its low cost and a physically flexible device. Wax can also be used as an ink in a pen by tracing out on a filter paper and heating the pattern in an oven [19]. Nie et al. described a one-step plotting method using a permanent marker and a metal mask for fabricating the device within 1 min [35]. Moreover, Chakraborty et al. demonstrated a single-step method for fabricating a µPAD using correction pens within 10 s [36]. As opposed to wax patterning, this method required no heating steps as the ink penetrates during the plotting. Although this method is straightforward, the thickness of the barrier is difficult to control. It is also important to note that both wax and commercial inks are unable to fully block the pores in the paper as hydrophobic materials, such as oil, will easily penetrate through these barriers because of their similar chemical nature.

**Inkjet Printing:** Wax printing and pen plotting are still in their initial stages in terms of commercialization. A technology that is commonly available in the market is inkjet printing. Hence, many researchers modified the inks and nozzles within the inkjet printer to fabricate μPADs and simplify the process for mass production [37]. One such chemical is AKD, which can selectively hydrophobize paper [38]. The technique involves printing AKD-heptane solution on paper and curing it at 100 °C for 8 min to create hydrophobic barriers. Although a simple technique, its disadvantage is the damage caused to the printer, especially to the cartridge, by organic solvents. The methods described by Maejima et al. and Citterio et al. used a UV-curable acrylate ink instead of the hazardous organic solvents for printing on paper; further, for curing, the pattern was exposed to UV light for 60 s [39,40]. Wang et al. also described an environmentally friendly method using hydrophobic sol–gel with inkjet printing or base etching to fabricate paper-based microfluidic devices [41]. Their pattern was able to endure surfactant solutions, glycerol, toluene, and dimethyl sulfoxide, which was not possible with wax and AKD. Recently, Citterio et al. demonstrated the usage of a simple desktop thermal inkjet printer for deposition of an assay reagent for a distance-based paper microfluidic device [42,43]. Inkjet printing has the advantage of using other reagents for sensing within the paper channels.

**Laser Printing:** Low-cost printing techniques, such as wax printing and inkjet printing, which have demonstrated a rapid process, have been developed; however, wax printers have not been commonly used and inkjet printers require a modification in the composition to fabricate a µPAD. Conversely, a laser printer demonstrated the advantage of a simple, high-resolution fabrication technique using a commercial device. Fu et al. printed patterns on both sides for electrodes on the µPAD using a laser printer [44]. Pushpavanam et al. also printed microchannel patterns on the paper using a commercial laser printer and original toner ink; the printed paper was heated at 165 °C to form a hydrophobic barrier [45].

**Flexographic Printing:** Another mass production method used in industries for printing on both paper and plastic is flexographic printing. In this method, polystyrene ink mixed in toluene or xylene is patterned using high-speed flexographic printing [46]. This technique can also pattern biomolecules, making the fabrication of a complete μPAD as a single step roll-to-roll process [47]. PDMS ink can also be flexographically printed onto copy paper; however, it requires six printed layers for a complete penetration into the paper compared to only one for inkjet printing [48]. The limitations of flexographic printing are in its specialized printing plates, multiple printing steps, and use of a single ink at a time, which hinders design flexibility. These limitations are countered by the high-throughput fabrication capability, which makes it a viable candidate for mass production.

**Stamping:** A rapid fabrication method using a PDMS-based stamp and an indelible ink for patterning onto a filter paper was described by Curto et al. [49]. The fabrication was achieved in less than 10 s by a one-time placement of the stamp on paper for 3 s, without requiring any cleanroom facility or washing steps. Although it is a simple and rapid method, the fabrication of the PDMS stamp is complex. Other similar methods used metallic stamp or foam stamp lithography to achieve the desired patterns [50,51,52]. The advantages of flash foam stamp are its widely used nature, easy fabrication, ink storage capability, and suitability for rapid prototyping in lab environments.

**Chemical Vapor Deposition:** Penetration into the thickness of the paper was achieved using chemical vapor deposition of hydrophobic photoresponsive poly(o-nitrobenzyl methacrylate) [53]. For the creation of hydrophilic regions, a black construction paper mask was cutout using laser cutter. After the coating, the paper was exposed to UV light and washed in a buffer to obtain the desired pattern. In another work, a poly(chloro-p-xylene) (PPX) film was patterned into paper using an evacuated sublimation chamber [54]. The hydrophilic regions were covered with the help of a metal mask held together by magnets on the top and bottom surfaces. Kim et al. used vinyl tape to cover the top of the paper before vapor deposition using chlorosilane [55]. In addition, a channel with chemical deposition was efficient in the control and delay of the liquid flow along with UV radiation time and channel geometry [56]. The advantage of this technique is that the hydrophilic channels are not affected by the solvents. However, chemical vapor deposition suffers from high-cost instrumentation, which is not easy to obtain in resource-limited settings.

**Wet Etching:** Cai et al. fabricated a μPAD by initially hydrophobizing paper with trimethoxyoctadecylsilane (TMOS) solution and then exposed it to a paper mask imbibed with NaOH solution for etching of the filter paper [57]. The masked area became hydrophilic while the unmasked area served as hydrophobic boundaries. The advantages of this method are that it does not require any costly instrumentation, metallic masks, and expensive reagents. The disadvantage is in using a paper mask, which suffers from low resolution.

**Hand-held Corona Treatment:** Jiang et al. used octadecyltrichlorosilane (OTS) to silanize paper to make it hydrophobic [58]. The paper was then washed with hexane and water and dried by nitrogen. Subsequently, it was clamped tightly between a polymethylmethacrylate (PMMA) mask and a soft pad and exposed to corona discharge at a low level to generate hydrophilic channels. The unexposed areas remained hydrophobic. This fabrication method is quick and cost effective for lab purposes; however, mass production will be complicated.

**Screen-printing:** Screen-printing method is used to transfer the ink onto a substrate using a patterned screen stencil and a squeegee. Hydrophobic materials, such as wax and polystyrene, are used as ink to create hydrophobic barriers for µPADs [59,60,61,62]. Moreover, Atabakhsh et al. used conductive paint to fabricate a heater or an electrode by using joule heating on the paper [63,64]. Lamas-Ardisana et al. used a mixture of UV-curable ink for forming hydrophobic barriers; carbon and silver/silver chloride inks were patterned over the hydrophilic areas for electrode systems [65,66]. This method has the advantages of low cost and simple fabrication steps; however, it demonstrates low resolution of microfluidic channels and rough barriers. Moreover, it is unadaptable for mass production as a different screen stencil is required to produce different designs.

**3D Printing:** Fu et al. used a 3D printing technique to fabricate a µPAD (Figure 2C) [67]. Initially, they printed a substrate for the microchannels, and PDMS was coated to seal the microgap caused by 3D printing. Then, the cellulose powder with deionized water was dispensed into the microchannels and dried in an oven. This fabrication method is significantly inexpensive, fast, and accessible to mass production by using a desktop 3D printer. He et al. used a desktop stereolithography 3D printer and a dynamic mask to fabricate μPADs [68]. In their method, the paper was first immersed in a UV resin followed by exposure to UV light through the dynamic mask. This was followed by curing to make hydrophobic barriers. The uncured regions were washed with anhydrous alcohol. The overall process drastically reduced the fabrication time to only 2 min.

**Spraying:** In another method, hydrophobic material was directly sprayed on paper covered with a mask, which created a hydrophobic barrier. Lew et al. sprayed the commercial water repellent product on a paper; moreover, the acrylic mask was manufactured using a milling machine mounted with a 500 µm diameter end mill [69]. Scholar glue, which is known as white glue, was sprayed on paper by Coltro et al. for the first time in 2017 (Figure 2D) [70]. This glue was sprayed using magnetic masks; subsequently, the paper was exposed to UV/Vis light from a halogen light source to form hydrophobic barriers promoted by crosslinking. This is an easy-to-use and equipment-free method for fabrication of µPAD; however, it demonstrates low resolution and uniformity when compared to other methods.

### 2.2. Physical Patterning

**Knife Plotter:** Fenton et al. employed a computer-controlled knife plotter for precise cutting of the paper to obtain the desired patterns [71,72]. To avoid the wrapping or tearing of paper, three sequential cuts were required. This simple cutting method reduces the fabrication time and can be scaled up to cut multiple devices in a single large sheet for high-volume manufacturing.

**Craft Cutting:** In a similar method, a digital craft cutter was used by Cassano and Fan to fabricate laminated paper-based analytical devices (LPAD) [73]. Paper was first attached to an adhesive sheet; subsequently, paper strips were cut using the craft cutter. Similarly, the covering and bottom sheets were also cut, assembled, and laminated to manufacture the LPAD. For a precise cutting of fragile and brittle paper, such as nitrocellulose, a sacrificial layer should be attached on top of the paper for a smooth cutout [73,74]. This also saves the material cost and time of fabrication. Glavan et al. used an omniphobic paper and a craft cutter to carve open channels on the surface of the paper [75]. The cut pattern was treated with fluoroalkyltrichlorosilane to make it omniphobic, followed by taping to seal the open channels. The advantages of this fabrication are its lightweight, flexible, portable, and disposable characteristics. However, unlike capillary flow in paper-based microfluidics, these devices require an external pumping mechanism; further, creating a variable width with a single blade is also difficult.

**Embossing:** Paper was dampened using ethanol and sandwiched between two plastic molds for embossing the desired pattern (Figure 2E) [76]. This was followed by silanization and then sealing with an adhesive tape to obtain the open channels with porous omniphobic walls. The authors described that the open channels could interact with the surrounding gases owing to the porous nature of paper, which is otherwise impossible. Additionally, flexible and foldable devices are also possible with this method.

**Laser Cutting:** Laser treatment of a hydrophobic paper surface helped create microfluidic patterns as small as 62 µm [77]. The process used a CO_2_ laser cutting machine to selectively cut and engrave on the paper surface, e.g., parchment, wax, or palette paper. During the engraving, the power and speed was optimized so that it does not cut through the entire thickness of the paper. The patterned regions were made hydrophilic by treating with silica micro particles.

Another CO_2_ laser cutting method used backed nitrocellulose membrane as the porous material to fabricate paper-based microfluidic devices [78]. The membrane was placed in-between two protective polymer sheets to avoid igniting the nitrocellulose, followed by ablation by a laser beam to achieve the desired patterns. The process is rapid and producible in both laboratory and industrial environments. The disadvantage of this method is that mass production at high throughput may not be possible as it requires multiple laser systems. Further simplifying the process, Nie et al. used a one-step cutting/engraving of paper using a minitype CO_2_ laser machine [79]. The paper was completely cut through its thickness to create a hollow microstructure-patterned paper, which behaved similar to the hydrophobic barriers. Bedin et al. fabricated the top and bottom layers using a 30W CO_2_ laser cutting machine for fluid inlets and flow, respectively; moreover, these two layers were stacked using an adhesive film (Figure 2F) [80]. Further, they controlled the thickness of the glass–fiber paper (bottom layer) by laser etching for fabricating a specific reaction area. The advantages of this method include rapid fabrication within 20 s, highly reproducible cutting, and a comparatively inexpensive laser cutting machine.

### 2.3. 3D Microfluidic Paper-Based Analytical Devices

The previously described techniques comprise the 2D μPADs, which perform satisfactorily for simple reactions in a planar surface. However, the addition of multiple reagents; improvement of mixing capabilities; addition of functional layers, such as filtration or integrated reservoirs; improvement of the speed of reactions; as well as sequential delivery of reagents is often difficult in a 2D surface. Hence, there is increasing interest in 3D μPADs for their superior analytical performance as well as the ability to offer smaller functional areas when compared to 2D μPADs.

**Figure 3:**
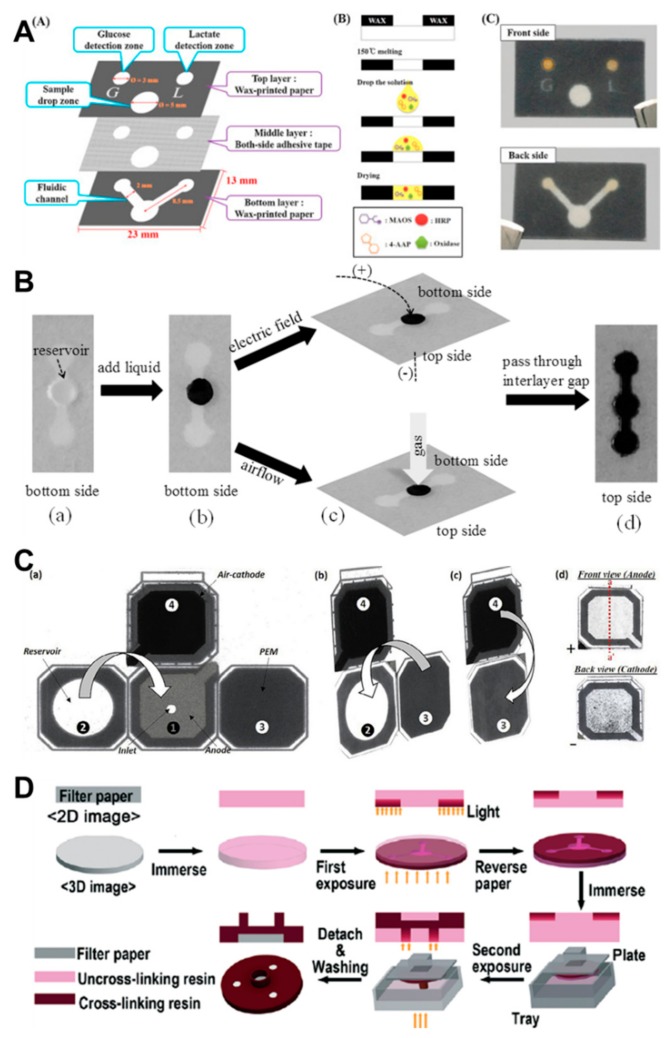
Three-dimensional paper-based microfluidic devices. (**A**) Stacking method using both-side adhesive tape (reprinted from ref [82] with permission from Elsevier, Copyright 2016), (**B**) 3D printer (reprinted from ref [88] with permission from Elsevier, Copyright 2019), (**C**) Origami method using wax-printed 2D paper sheets (reprinted from ref [95] with permission from Elsevier, Copyright 2017), (**D**) Double-sided 3D printing method using digital light processing printer (reproduced from ref [100] with permission from the Royal Society of Chemistry. 2018).

**Stacking:** The traditional fabrication technique of a 3D µPAD involves stacking of layers of patterned paper by diverse 2D fabrication techniques, such as wax and screen-printing techniques, and cutting with a double-sided adhesive tape (Figure 3A) [81,82,83,84]. The direction of fluid flow is not only horizontal but also vertical. However, the stacking method has limitations of complicated alignment, bonding, and punching processes. Therefore, Li et al. and Jeong et al. demonstrated a partial double-side printing and lamination method to fabricate a 3D µPAD with a single layer [85,86]. Moreover, the stacking method was extended to integrate with other methods, and Chang et al. used a cut-and-insert method, where the central region of two papers were cut and inserted to overlap and connect the fluid flow [87]. This method could transfer the fluid more easily and freely as required. Furthermore, the stacking method was applied in combination with a 3D-printing technique; the resin of the 3D printer was cured between the building platform and release paper along with the print design (Figure 3B) [88].

**Origami:** Origami is one of the inherent characteristics of paper and can be applied to various paper-based devices [89,90,91,92]. Qin et al. originally fabricated a 3D µPAD by integrating with a solid-contact ion-selective electrode and an all-solid-state reference electrode in a 3D origami device [93]. The wax printing method was used to form a hydrophobic barrier for fluid flow on the 2D sheet papers, which were folded for different operations (Figure 3C) [94,95,96]. Additionally, a paper mask coated with PDMS was used to fabricate a contact-printed µPAD using a folded paper [97]. This method demonstrated a simple operation by folding paper, where the fabrication process was fast, easy, flexible, and low cost. Moreover, the 3D structure could control timing and effectively reduced the problem of solution diffusion by using a lateral flow strip.

**Open-channel:** Recently, surface flow by manipulating the surface wettability has been increasingly investigated as an alternative to the conventional capillary driven flow through the porous media, which is limited by the slow wicking [15]. The flow dynamics are governed by the fluid interface, which is dependent on the channel geometry and the chemical composition of the surface [56]. Recent advances in this open-channel microfluidic device using paper have enabled the flow of not only water but also low surface tension organic liquids allowing complex applications, such as emulsification in open channel format [98]. Transferring this into the 3D format, Li et al. developed an open channel paper-based platform capable of manipulating as well as transporting all high and low-surface tension liquids using fluoro-silanization followed by a masked O_2_ plasma treatment with varying timings for selective etching [99]. Their device was perforated to permit fluid flow through the thickness of the paper to the other side. The 3D device increases the interfacial area and enables fabrication of complex assays on a single μPAD.

**3D Printing:** Park et al. fabricated 3D µPAD using a double-sided 3D printing method (Figure 3D) [100]. They used a digital light processing-based 3D printer, which formed a hydrophobic barrier by exposing photocurable polymers. The filter paper was immersed in a photocurable resin and initially exposed to visible light from the printer. After the first exposure, the other side of the paper was re-exposed to form the covering and sample reservoir. Finally, the paper was washed with ethanol to eliminate the remaining resin. This method could be mass produced with relatively easy and simple processes. In addition, Nie et al. used filter paper and pulp mud to fabricate the modular microfluidic devices. 3D printer manufactured the frame of the module, and PDMS was used for surface treatment, then capillary materials were filled and dried. This method demonstrated a modular system using 3D printing and paper, therefore base modules could be simply assembled together for the specific usage such as 3D direction flow and diverse height channels [101].

In summary, both 2D and 3D fabrication methods have seen significant improvements for specific applications. However, more work needs to be done to produce a universal platform for a variety of applications. Additionally, cost-effective and simple fabrication methods with high resolution need to be developed for better performance and commercialization of μPADs. The simplicity of the wax-printed or screen-printed devices could be used for resource-limited settings where the high throughput or reproducibility of the device are not major issues.

## 3. Fluid Control and Handling

### 3.1. Theory

Understanding the flow behavior is vital for the development of a highly accurate, predictable, and programmable paper-based microfluidic device. It is possible to control or manipulate fluid velocity for sequential delivery only after all the control parameters affecting the fluid flow are known. We understand that the flow in paper is governed by the capillary flow at a low Reynolds number, which hinders the mixing capabilities in straight channels. In the upcoming section, we discuss the dry flow or paper wet-out, fully wetted flow, and various geometrical effects governing the flow rate.

**Paper Wet-out**: Paper wet-out is a flow condition where the fluid comes into contact with the porous media for the first time. Wicking in paper is at low Reynolds number; hence, it is categorized into the laminar flow regime [8,102].

The fluid flow in paper is often modeled by the Lucas–Washburn equation, derived from the combination of capillary theory and Hagen–Poiseuille’s law, which predicts that the length of travel *l* for a 1D flow is proportional to the square root of time *t* as predicted in Equation (1) below [103].

(1) $$l=\sqrt{\frac{\gamma rcos\theta t}{2\mu }}$$

It includes the surface tension of the liquid γ, the average pore radius which is also called as effective pore radius *r*, and the contact angle θ between the fluid and the boundary wall. Although an effective solution, the Equation (1) has many limitations. First, it can only be used for 1D flow, which implies that a variable cross-sectional flow area will deviate from this equation. Second, it assumes negligible gravitation and evaporation effects and a continuous supply of reservoir. Third, it ignores the effects of hydrophobic boundaries on the capillary flow. Additionally, it also does not consider micro-scale intra-fiber flow which also affects the fluid flow profile. A few of these conditions are discussed below with modified or new equations to predict fluid flow in paper-based microfluidic devices.

**Fully-wetted Flow:** For a pre-wetted paper, the fluid flow for a constant cross-section can be modeled using Darcy’s law [104]:

(2) $$Q=-\frac{kA\Delta P}{\mu l_{0}}$$

In Equation (2) *Q* represents the volumetric flow rate, *A* is the cross-sectional area perpendicular to the flow, k represents the permeability of the paper, and ΔP is the pressure difference across $l_{0}$. Darcy’s law comes from the Navier–Stokes equation and assumes straight capillaries and circular fiber cross-section.

**Different Channel Width Segments:** The Equation (2) can also be applied to determine the flow rate for varying cross-sections. This can be achieved by imposing equal volumetric fluxes in straight channel segments connected to each other but having different widths [105]. The Darcy’s law is modified to give the flow rate is given by:

(3) $$Q=-\frac{\Delta P}{\left(\frac{\mu }{k}\sum_{i=1}^{N}\frac{l_{i}}{w_{i}b_{i}}\right)}$$

Here, $w_{i}b_{i}$ represent the channel segment cross-sectional area. For a paper having N number of channel segments, the total volumetric flux can be determined using the Ohm’s law analogy for electrical circuits. The voltage and current are replaced by the pressure difference ΔP and flow rate *Q*. The fluidic resistance of each segment *i* is given by $\frac{\mu l_{i}}{kw_{i}b_{i}}$. For a fully-wetted flow, the velocity for a constant width is constant. For a series of different widths, the resistances can be added in Equation (3) to determine the velocity.

**Continuously Increasing Channel Width:** When a fluid encounters a continuously increasing channel width, such as a circular sector, the fluid front advances radially as the bed volume is continuously increasing. For such fan-shaped sectors, the fluid velocity is constant when observed through a rectangular segment of width $w_{r}$ [106]. This quasi-steady flow is predicted by Equation (4) below:

(4) $$Q\approx \frac{k_{i}w_{r}P_{c}}{\mu l_{r}}$$

(5) $$P_{c}=\frac{\mu }{k_{i}}r_{f}{\dot{r}}_{f}\left(\frac{l_{r}\omega }{w_{r}}+\ln\left(\frac{2r_{f}\left(t\right)}{w_{r}}\right)\right)$$

Here, $l_{r}$ is the length of the rectangular segment, $P_{c}$ is the capillary pressure for a fan shape (Equation (5)), $r_{f}$ is the radial distance of flow, ${\dot{r}}_{f}$ is the radial velocity, ω is the central angle, and $k_{i}$ is the interstitial permeability. The fan-shaped sectors of decreasing angles can also be joined together to attain a constant flow velocity when observed in a linear 1D flow [74].

**General Equation for Arbitrary Cross-section:** A general equation for any kind of cross-sectional area can be derived using a macroscopic approach. Here, the local flow in small pores can be overlooked. The flow is assumed to be stationary and free of inertia with gravity ignored. Under the Stokes regime, the continuum fields for the fluid velocity and pressure follow the equation given below [107]:

(6) ∇⋅u=0

(7) $$u=-\frac{k}{\mu }\nabla P$$

Equation (6) is the mass conservation which considers an incompressible fluid with no change in evaporation or condensation. Equation (7) is the force-flux relationship for linear dimension in porous media also defined previously in Equation (2). Considering a varying cross-sectional area $A\left(x\right)$ along the flow, the Equation (7) can be integrated by substituting $Q=A\left(x\right)u\left(x\right)$ as imposed by a continuity equation at an instant, from pressure *P_o_* at x = 0 and capillary pressure *P_c_*:

(8) $$P_{0}-P_{c}=Q\frac{\mu }{k}\int_{0}^{l}dx/A\left(x\right)$$

In Equation (8), the capillary pressure is given by the Laplace equation ($p_{c}=p_{atm}-2\gamma \cos\theta /r$). By defining a generic flow resistance $R_{0}=(P_{atm}-P_{0})/Q$, and $∆P=P_{atm}-P_{c}$, the equations are added to obtain the fluid front velocity:

(9) $$u\left(l\right)=\frac{∆P}{A\left(l\right)\left[R_{0}+\frac{\mu }{k}\int_{0}^{l}\frac{dx}{A\left(x\right)}\right]}$$

In Equation (9), $A\left(l\right)$ is the cross-sectional area corresponding to the fluid front velocity $u\left(l\right)$. As $u\left(l\right)=dl/dt$, integrating this into the Equation (9) reveals the implicit expression for the fluid front position l against time t:

(10) $$\frac{kR_{0}}{\mu }\int_{0}^{l}A\left(l^{\prime }\right)dl^{\prime }+\int_{0}^{l}\left[A\left(l^{\prime }\right)\int_{0}^{l^{\prime }}\frac{dx}{A\left(x\right)}\right]dl^{\prime }=Dt$$

Here, *D* (m^2^/s) is similar to the diffusive coefficient (D=k∆P/μ). Equation (10) can predict the time for filling any arbitrary shape of paper, whether it is 1D or 2D, and inversely the fluid velocity can be used to obtain the cross-sectional profile.

**Effect of Hydrophobic Barriers:** The effect of a wax boundary on fluid flow was studied by Hong and Kim [108]. The hydrophobic wax on the channel sides result in a higher contact angle as opposed to the bulk capillary flow. Hence, a resistance force is added to the overall force balance over a control volume as:

(11) $$\left(\pi d\gamma cos\theta -8\pi \mu ldl^{\prime }\right)\left(\frac{w}{d}\phi^{\frac{1}{3}}\right)\left(\frac{b}{d}\phi^{\frac{1}{3}}\right)+\beta \pi d\gamma cos\theta_{b}\left(\frac{b}{d}\phi^{\frac{1}{3}}\right)=0$$

Here, β is the length of the advancing contact angle lines, which is in contact with the hydrophobic wall and should be determined by experimentation; w and b are the width and thickness of the channel, respectively, ϕ is porosity, and d is pore diameter. Simplifying and solving the Equation (11), the capillary flow bounded by hydrophobic wax can be modeled by:

(12) $$l_{b}\left(t\right)=c\sqrt{\left(1+\beta \frac{dcos\theta_{b}}{\phi^{\frac{1}{3}}wcos\theta }\right)\frac{\gamma }{\mu }t}$$

The Equation (12) describes the imbibition length $l_{m}$ with respect to time *t* with a proportionality constant *c*, which defines the geometry of the pores depending on pore radius and contact angle. The model was experimentally validated with a variety of boundary forms and it also provides a method to manipulate the fluid velocity by using the hydrophobic drag. Additionally, it is important to note that this study is regarding the flow of water against a hydrophobic wall. While considering the effect of similar natured fluids on the boundary, such as oil flow in a hydrophobic channel, the effect of channel wall can be ignored [109].

**Effect of Evaporation:** Liu et al. developed a model by incorporating the effects of evaporation in addition to the capillary driven flow with viscous resistance [110]. The calculation was based on predicting the evaporation rate $m_{ev}^{*}$ from water saturated pressure, latent heat of vaporization of water, air flowrate, and relative humidity, and using it to determine the wetted length $l_{ev1}$ by the following first order differential equation:

(13) $$l_{ev1}=2Ne^{-Mt}\int_{0}^{\sqrt{t}}e^{Mt^{2}}dt$$

In Equation (13), $M=\frac{2\;m_{ev}^{*}}{\rho \varepsilon b}$ and $N=\sqrt{\frac{\gamma \cos\theta }{\mu }\frac{k}{\varepsilon r}}$ with ρ as density, and ε as effective porosity.

Camplisson et al. also took into account fluid evaporation in Equation (1) to determine the wetted length $l_{ev2}$ by [111]:

(14) $$l_{ev2}=\sqrt{\frac{\gamma r\phi b\cos\theta }{4\mu q_{0}}\left(1-e^{-2q_{0}t/\phi b}\right)}$$

In Equation (14), $q_{0}$ is the rate of evaporation determined from the volumetric rate of liquid evaporation per unit area.

A more recent work on the Lucas-Washburn equation was performed by incorporating effects of different relative humidity by Castro et al. [112]. The effects of water saturation $S_{w}$ and evaporation are characterized by modifying the evaporation model of Fries et al. [113]. The water saturation model predicts the wetted length $l_{ev3}$ in paper by Equation (15):

(15) $$l_{ev3}=\sqrt{\frac{a_{1}-a_{1}exp\left(-2a_{2}t\right)}{a_{2}}}$$

(16) $$a_{1}={\left(\frac{k_{e}}{2\phi }\right)}^{1/2}\frac{\gamma \cos\theta }{\mu }\;a_{2}=\frac{F\left(w+b\right)}{\phi \rho \left(1-S_{w}\right)wb}$$

In Equation (16), $k_{e}$ is effective permeability, and F is the evaporation flux. In case of F=0, i.e., neglecting evaporation effect, the Equation (15) reduces to the Lucas-Washburn Equation (1).

### 3.2. Flow Control Methods

Recently, several advancements were achieved in the field of microfluidics that permitted a controlled fluid flow and performed multistep reactions on paper. These methods are valve-like control tools, which enable the delivery of accurate quantities of chemicals with impeccable timing. The controlled transport of reagents resulted in the possibility of multi-analyte detection in paper-based microfluidic devices, which eventually leads to complex reactions, such as enzyme-linked immunosorbent assay (ELISA) to be performed on paper networks. These methods can be categorized into geometry-, chemical-, and mechanical-based methods.

#### 3.2.1. Geometry-Based Methods

**Figure 4:**
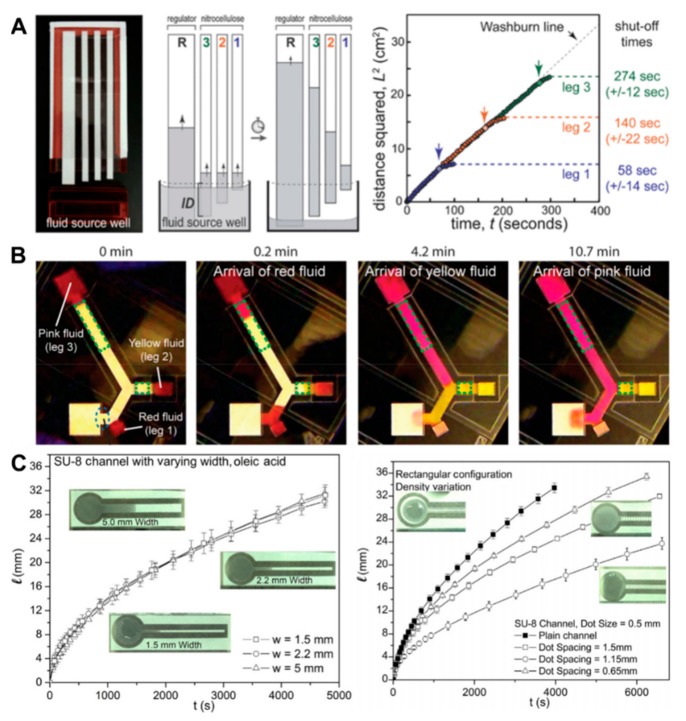
Flow control using geometry-based methods. (**A**) Three nitrocellulose membranes with different lengths are dipped in a volume-limited well to control flow rate and disconnection from the reservoir (reproduced from ref [115] with permission from the Royal Society of Chemistry 2011). (**B**) Images of sequentially delivered red-, yellow-, and pink-dyed fluids using cellulose shunts (reprinted with permission from ref [119]. Copyright 2013 American Chemical Society). (**C**) Flow comparison of oleic acid along with width and dot density variation (reprinted from ref [109] with permission from Elsevier, Copyright 2016).

Geometry-based methods achieve a flow rate control through an alteration of the fluid channel geometry. This includes variations in channel length, channel width, or flow path that can alter the flow velocity. Fu et al. controlled the length of travel to perform a multistep reaction in a 2D paper network [114]. The three inlets having different reagents were spaced with increasing distances from the detection zone, each having a higher volume depending on the length of travel. By controlling the flow rate, they showed chemical amplification and therefore higher sensitivity, which is otherwise not possible in paper channels. This technique was improved by employing a variable leg length that was dipped in a single buffer source (Figure 4A) [115]. After the required volume of fluid was transported through the first leg, the source was disconnected because of the drop in the height of the buffer. This enabled both controlled flow rate as well as controlled shut off from the reservoir of each reagent. Fast flow was first reported by Jahanshahi-Anbuhi et al. by placing flexible films at the upper and lower surfaces of the paper channel [116]. This method resulted in an accelerated flow by forming a wedge at the liquid front, which obeys the law that the height of travel is proportional to the cube root of time. Channon et al. fabricated a rapid flow rate multilayered device by increasing the channel height to 390 µm and sealing it at the top and bottom with packing or double-sided tape [117]. They achieved a 145-fold increase in flow rate when compared to the simple lateral flow in µPADs. Subsequently, the group further quantified this flow through narrow gaps by making a gap between two paper channels and covering both sides with packing tape [118]. It was determined that the flow was initially governed by Laplace pressure and afterwards it was driven between the porous channel walls as well as into these walls simultaneously with a convex/concave fluid front. Toley et al. demonstrated the use of an absorption pad to produce a delay in fluid flow, which can be adjusted by varying pad thickness and size (Figure 4B) [119]. A fan-shaped membrane used by Mendez et al. even permitted quasi-stationary flow in the fluid channels as the membranes replaced the wicking regions on paper [106]. This flow deviated from the Washburn law as it resulted in a linear length–time relationship in a 1D flow. Jafry et al. demonstrated the use of a tortuous flow path in a paper-channel to vary the flow velocity (Figure 4C) [109]. Circular obstacles or cylinders within a paper-channel resulted in increased resistance to flow and by varying their configurations, we achieved different flow rates without changing the length or width of the channel. Similarly, wax-printed horizontal lines or barriers in a straight channel caused the flow to travel through a tortuous path resulting in a delay to reach the detection zone [22]. A trapezoid-shaped geometry was designed in this case for delayed and non-delayed channels facilitating flow speed, uniform division, merging of samples, and eventually improved sensitivity of the device. In a similar attempt to enhance the generated signal, a trapezoid constriction in a lateral flow immunoassay device improved the analytical performance by slowing down the fluid flow rate as well as concentrating the analyte molecules to pass through a constricted zone [120].

#### 3.2.2. Chemical-Based Methods

**Figure 5:**
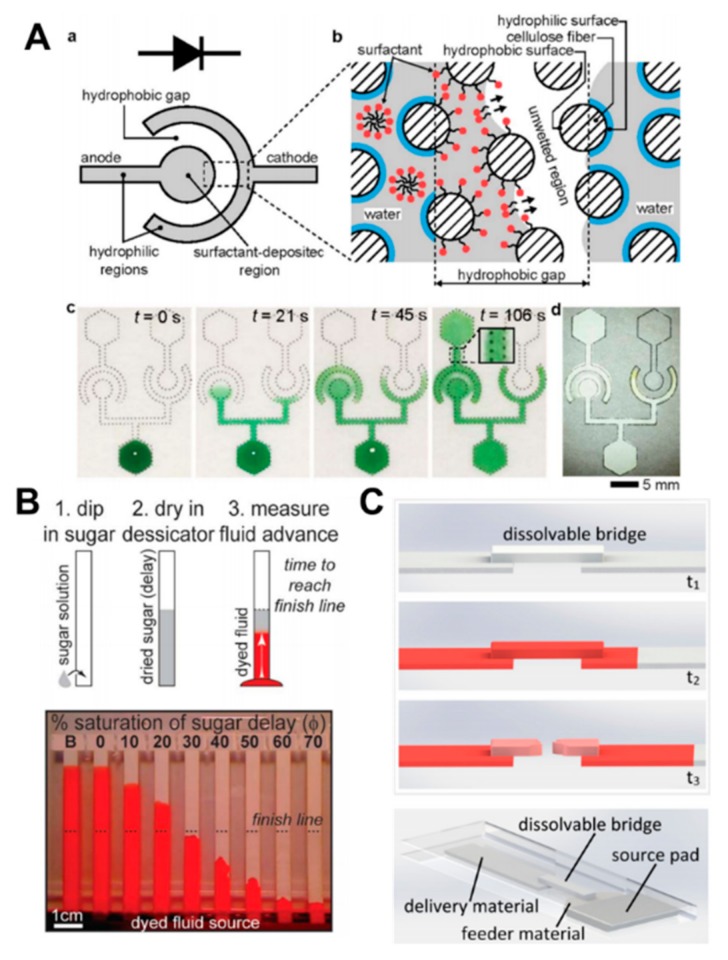
Flow control using chemical-based methods. (**A**) Schematic of a one-directional fluidic diode and illustration of the working mechanism of the fluidic diode. Fluid flows through the hydrophobic gap from the surfactant side. The images show that the green-dyed fluid flows through the diodes (reproduced from ref [121] with permission from the Royal Society of Chemistry, 2012). (**B**) Preparation steps of delayed strip using sugar solution. Experimental images show the flow test with varying concentrations of sugar solution, and the dashed line and strip “B” indicate the finish line and an untreated strip, respectively (reproduced from ref [122] with permission from the Royal Society of Chemistry, 2013). (**C**) Schematic of operation mechanism and set-up of a dissolvable bridge. The bridge dissolves at a certain time to shut-off the fluid flow (reprinted with permission form ref [124]. Copyright 2013 American Chemical Society).

Chemical methods utilize various chemicals embedded in the channel that cause variation in flow rates or disconnection of the supply from reservoir to stop the flow. Noh and Phillips deposited paraffin solution in hexane into small regions of patterned paper to meter the flow rate of fluids in a 3D paper-channel [7]. Chen et al. developed a one-directional fluidic diode in which a hydrophobic barrier was sandwiched between hydrophilic regions, one of them containing a surfactant (Figure 5A) [121]; fluid crossed the hydrophobic barrier from the surfactant side. Another single use on the valve utilized OTS solution in paper to make it hydrophobic and then exposed it to UV light [58]. The exposed region turned hydrophilic, permitting the fluid to pass. Lutz et al. introduced flow delays by dissolving a varying amount of sugar into the paper-channels resulting in a multistep fluidic protocol (Figure 5B) [122]. Inkjet printing of conductive hydrophobic and hydrophilic electrodes and applying electric potential between them caused a delay in the fluid flow similar to a valving mechanism [123]. This principle was based on electrowetting on dielectrics, which polarizes a hydrophobic electrode into a hydrophilic one. Another valving mechanism to manipulate fluid flow used dissolvable bridges within a paper channel to tune the volume of circulated fluid (Figure 5C) [124]. The bridge dissolves after a certain amount of fluid travels across, resulting in an automated delivery from multiple resources. A similar method used water-soluble pullulan as the dissolvable polymer to function as an automated shutoff valve for multistep reactions [125]. However, the disadvantage of these methods is that the paper channel or bridge is composed of chemicals, which may limit the use of certain reagents that are incompatible with the added chemical. An attempt to overcome these limitations was performed by Strong et al. by fabrication of wax-printed fluidic time delays on the top and/or bottom layers of paper by varying the degree of channel coverage permitting a range of flow rates [126]. By integrating the valving functionality into wax-printed devices, a potential POC device can be created that is cost effective, simple, and reliable with the capability to conduct complex multistep assays with relative ease. Chen et al. developed a wax-based valve that operated on the principle of dissolving the printed wax by organic solvents and opening the channel for fluid flow [127].

#### 3.2.3. Mechanical-Based Methods

**Figure 6:**
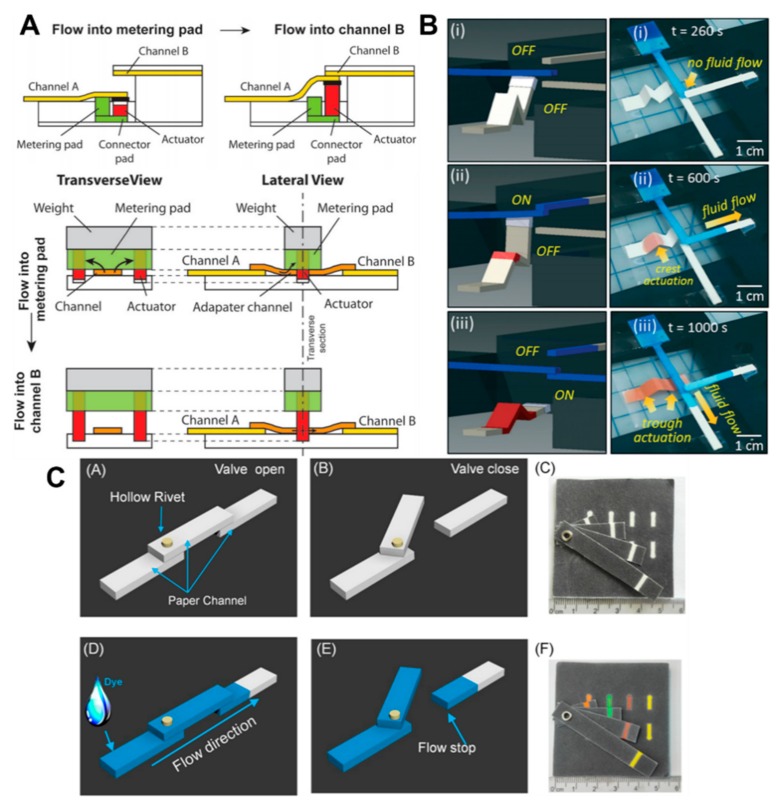
Flow control using mechanical-based methods. (**A**) Schematic of a volume-metered valve with a cantilever channel and moveable metering pad. In a cantilever channel, when the actuator is expended by the delivered fluid from the metering and connector pads, channels A and B are connected. In a moveable metering pad, as the solution flows through the metering pad, the actuator swells up to form space; then, the fluid flows into channel B (reproduced from ref [128] with permission from the Royal Society of Chemistry, 2015). (**B**) (**i**) Only blue dyed water flows through channel owing to the physical separation from other channels. (**ii**) When the red dyed water is dropped on the “W” shaped actuator, the tip of the actuator connects with the primary channel and the blue dyed water flows through the upper channel. (**iii**) The tip of the actuator connects with the bottom channel after trough actuation (reproduced from ref [129] with permission from the Royal Society of Chemistry 2017). (**C**) Schematics and experimental figures of the hollow-rivet-assisted movable valve paper device. The valve opens and closes as the movable channel rotates around the pivot (reprinted with permission from ref [130]. Copyright 2017 American Chemical Society).

Mechanical methods involve physical motion of components that permit connection, disconnection, or close proximity of the channel surface. Mechanical switches to change fluid flow through channels, developed first by Toley et al., is perhaps the most versatile method of fluid control (Figure 6A) [128]. The valve actuates after a certain time interval or with the passage of a specified fluid volume. The mechanism requires the displacement of one end of the channel, causing new connections to be created with downstream channels or preexisting ones to be broken. Kong et al. [129] used a folded chromatography paper, which was actuated by fluid addition at critical places, while Li et al. [130] used hollow rivets as hinges to unite or break up paper channels (Figure 6B,C). Another valving mechanism, similar to a desk calendar, was designed using plastic comb binding spines [131]. The ring-shaped binders can be simply flipped over to help connect the channels when required. A rotational valve was also used to connect the detection zones with the flow channel, which helped in reducing the detection error [132]. Moreover, Matsuda et al. demonstrated an active valve by an inkjet-printed heater using AgNP ink with filtered size of 5 µm [133]. The temperature of the heater reached 60 °C in 40 s with 0.75 mm printed width. The heating effect caused the fluid to evaporate, stopping the flow at the point. Once the heater was turned off, the fluid continued to flow. Phillips et al. also utilized heating by a polyimide thin-film heater to actuate valves using wax-printed patterns [134]. The wax-printed width of 0.1 mm on nitrocellulose membrane was demonstrated as multiple actuation of open, close and re-open of the same valve by simply heating at the valve position for specific time intervals.

In summary, much research is yet to be done for enabling a precise fluid flow control in μPADs. Current research is either difficult to fabricate at mass scale, or has a complicated protocol with expensive materials. Sealing of the device also needs to be taken into account for avoiding environmental contamination and evaporation effects which in turn leads to improved flow control. Hence, a solution involving low-cost flow manipulation, with an inexpensive fabrication technique, improved packaging, and simple to use platform would result in a successful point-of-care device.

## 4. Detection Techniques and Applications

The primary aim of paper-based microfluidic devices is to provide a user-friendly, cost-effective, and portable diagnostic tool for the end consumer. To achieve mass production of diagnostic assays, the product cost must be minimized; further, simple fabrication techniques are required for commercial success. Currently, μPADs are the best alternative among the devices being used in the industry today.

The major applications for paper-based microfluidics are medical diagnostics, environmental monitoring, and food quality control [6]. To detect the analytes in these potential areas, suitable transduction techniques need to be developed and optimized. These include colorimetric [135,136,137,138,139], electrochemical [140,141,142], chemiluminescence [143,144,145,146,147], electrochemiluminescence [148,149,150,151,152,153,154], and fluorescence [155,156,157,158,159,160] detection techniques. These techniques permit a simple, reliable, miniaturized, and cost-effective detection for various application areas. In the subsequent section, we have elaborated on each detection technique.

### 4.1. Colorimetric

**Figure 7:**
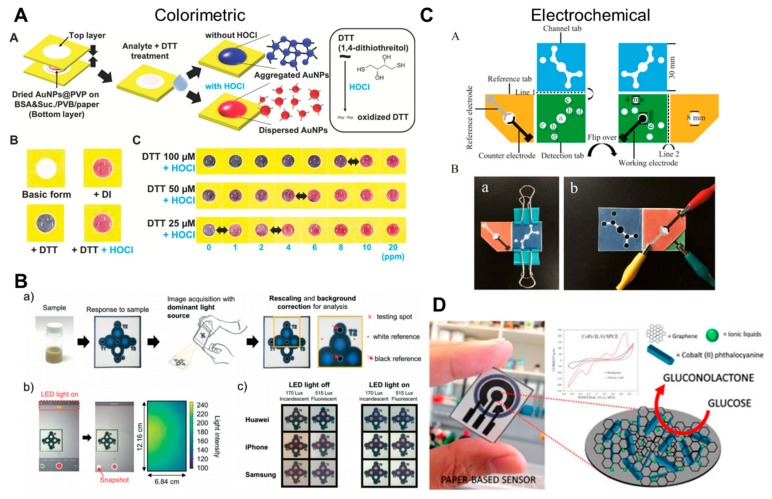
Detection techniques and recent applications of μPADs. (**A**) Colorimetric sensing of HOCl via AuNPs by controlling the concentration of dithiothreitol (reprinted with permission from ref [139]. Copyright 2019, WILEY). (**B**) Colorimetric sensing using a smartphone with integrated light source (reproduced from ref [172] with permission from the Royal Society of Chemistry, 2019). (**C**) Electrochemical detection of microRNA with chromogenic reaction (reprinted from ref [178] with permission from Elsevier, Copyright 2018). (**D**) Non-enzymatic electrochemical glucose sensing with cobalt phthalocyanine, graphene, and ionic liquid (reprinted from ref [177] with permission from Elsevier, Copyright 2017).

Colorimetric detection is one of the most commonly used detection techniques, which also includes the pregnancy test strip devised using lateral flow assays. It involves visually observing the color change during a reaction and using it for either qualitative or quantitative analysis with the help of the naked eye or a visual aid. Colorimetric detection offers the most simple and frequently used detection in the areas of protein analysis [36,161,162,163,164], drug analysis [23,165,166,167], and ion detection [40,42,139,168,169,170,171], covering all the fields from biomedical assay to environmental monitoring as well as food safety discernment (Figure 7A).

As camera phones are now integrated with several types of colorimetric readout software, their usage in resource-limited settings is now possible (Figure 7B) [81,82,172]. Using a permanent marker, a paper-based microfluidic chip can be fabricated for the colorimetric detection of nitrite ions. The color change is easily visible, and the signals are recorded by a camera phone and the results quantified for determining the concentration [163]. Moreover, detection of Fe^2+^ and Cu^2+^ ions is also possible using a smart phone camera and an RGB color reader application [171]. Wu et al. used an electrokinetic mechanism to concentrate and separate charged analytes in a paper channel. Two different food dyes (brilliant blue and amaranth) were detected and analyzed on the paper-based field-amplified sample stacking device in addition to bovine hemoglobin and cytochrome c by relying on their different electrophoretic mobility and using a cellphone camera for signal readout [173].

Owing to its simplicity, not only single components but multiple analytes can be detected simultaneously [70]. A paper-based assay was developed with a target-responsive aptamer cross-linked hydrogel for detection of multiple analytes, such as cocaine, adenosine, and Pb^2+^ [165]. The hydrogel is formed in the absence of a target, stopping the flow and hence the signal. When the target is present, no hydrogel is formed and the indicator travels to the detection region for a colorimetric readout. In addition, a chemically-patterned µPAD can detect various biomolecules, such as glucose and tumor necrosis factor alpha, as well as heavy metal owing to the immobilizing amine functional group using thermal condensation [55].

The advantages of colorimetric sensing are its low cost, simple fabrication, and rapid detection of multiple analytes simultaneously with the naked eye of a cell phone camera. Its disadvantages include lower sensitivity for a colorimetric readout in the visible range; moreover, the background paper color or lighting can cause problems in the automated readout.

### 4.2. Electrochemical

Electrochemical detection involves the direct conversion of a biological or chemical signal into an electrical one. Although fabricating a sensitive electrode results in a marginally expensive device when compared to the colorimetric sensing; electrochemical detection offers compact, accurate, highly sensitive, and selective platforms, which are otherwise not possible. Future advances in printing techniques and reduction in the cost of NPs or conductive inks will further reduce its cost creating a viable and attractive option. Dungchai et al. reported a screen-printing method to create electrodes for the accurate detection of glucose, lactate, and uric acid [174]. As oxidase enzyme produced hydrogen peroxide while decomposing their substrates, only a single electrode was required to detect multiple species. Cancer biomarkers are difficult to detect owing to their low concentrations. Wu et al. reported a signal amplification strategy in μPADs by incorporating graphene to accelerate electron transfer and using silica NPs for labeling signal antibodies [175].

Additionally, 3D devices having hollow channels were also proposed using a “Y” shaped channel design [176]. The results are obtained after the flow stops and the working electrode can determine the composition of each of the two streams independently. Furthermore, multi-layered paper-based sensors were fabricated for non-enzymatic glucose and microRNA (Figure 7C,D) [177,178]. These devices operate within a linear range of concentration of target molecules and have a low detection limit.

Electrochemical detection depends upon the electrode material, different modes, such as potential measurement [179,180,181] and amperometric detection [142,182,183] for analysis.

The disadvantages of this technique include a conductivity problem while using a poor conductive material, electrical continuity of the circuit on paper because of its flexible nature, and the difficulty in the mass production of highly-sensitive electrodes.

### 4.3. Chemiluminescent and Electrochemiluminescent

**Figure 8:**
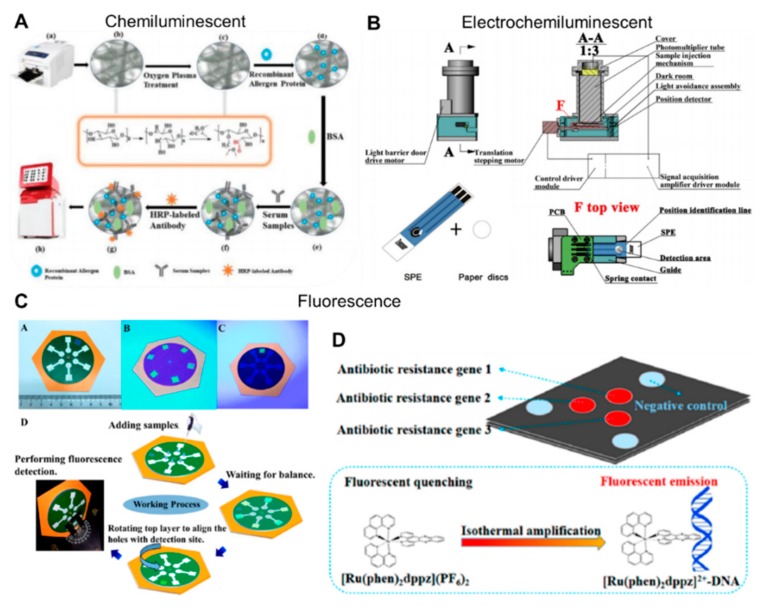
Detection techniques and recent applications of µPADs. (**A**) Chemiluminescent detection of paper-based immunoassay using horseradish peroxidase (HRP)-labeled antibody (reproduced from ref [188] with permission from the Royal Society of Chemistry, 2019). (**B**) Electrochemiluminescent detection of the antigen of hepatitis B virus from clinical serum samples (reprinted from ref [195] with permission from Elsevier, Copyright 2017). (**C**) Fluorescent detection of phenolic pollutants using quantum dots (reprinted with permission from ref [202]. Copyright 2018 American Chemical Society). (**D**) Antibiotic resistance gene detection via fluorescence sensing using a light source (reprinted with permission from [203]. Copyright 2018 American Chemical Society).

Chemiluminescence (CL) measures the light intensity from a chemical reaction. It provides advantages of cost-effective reagents, higher sensitivity, and less equipment cost. Examples of this detection method include determining glucose concentration in tears [184], dichlorvos measurement in vegetables without the interference of metal ions or vitamins [185,186], analysis of long DNA amplicons by hybridization reactions [187], and determining specific antigens by using the antigen–antibody reactions (Figure 8A) [188]. CL is also recognized for its high sensitivity. For instance, ofloxacin detection was performed on a sensitive wax-printed paper with luminal, where its intensity was increased by AgNPs [189]. This resulted in a highly-sensitive device capable of detecting up to 3.0 × 10^−10^ g/mL.

Electrochemiluminescence (ECL) produces measurable light intensity during an electrochemical reaction. This technique also provides an attractive substitute to the other detection techniques. Yu et al. developed paper-based ECL signal amplification devices using 3D origami technique for enhanced detection of proteins [190,191]. Wu et al. fabricated a microfluidic paper-based ECL origami cyto-device in which aptamer-modified Au electrodes were used as a working electrode for cancer cells capture and cyto-sensing [192]. Additionally, a bipolar electrode (BPE) array was fabricated for ECL detection of pathogenic DNA [193]. This paper-based BPE device had 15 units, each having six sensing and two reporting cells designed by wax. The sensor array successfully demonstrated multiplexed analysis of the syphilis (Treponema pallidum) gene, HIV, and hepatitis B virus gene. Xu et al. screen-printed electrodes on poly(sodium 4-styrenesulfonate)-functionalized graphene [194]. The produced assay was highly sensitive with long-term stability. Moreover, Shan et al. fabricated the paper-based ECL biosensing platform with a custom-made device for detecting the surface antigen of the hepatitis B virus in real clinical serum samples and demonstrated improved detection results when compared to commercial sensors, such as enzyme-linked immunosorbent assay and chemiluminescent immunoassay (Figure 8B) [195].

The disadvantages of CL and ECL include complicated device fabrication or amplification strategies, time-consuming method, higher cost, and difficulty in appropriately generalizing the luminescence signals in all situations.

### 4.4. Fluorescence

Fluorescence, unlike chemiluminescence, depends on external excitation by light or electromagnetic radiation, and in response, the object emits lower energy radiation usually in the visible spectrum. The light intensity is then quantified for the measurement of analyte concentration. Recent advances include escherichia coli growth quantification using a fluorescent mCherry label with the help of smart phone or a flatbed scanner, detecting values as low as 1–10 colony-forming units in 100 µL [196]. Rosa et al. demonstrated the capture and detection of fluorescein-labeled DNA on paper using the high binding affinity of Clostridium thermocellum to cellulose [197]. Thom et al. developed a galvanic fluidic battery to power a UV light-emitting diode within a 3D μPAD, which helped in the detection of a β-D-galactosidase fluorescent assay [155]. Yamada et al. detected lactoferrin in human tears by inkjet printing of terbium onto the paper surface, reaching a lower detection limit of 0.3 mg/mL [156]. The μPAD was excited by hand-held UV lamps and emitted a green fluorescent signal, which was filtered using a long-pass filter and captured by a digital camera. Another work measured the fluorescent intensity of paper modified with DNA-conjugated microgels to achieve a detection limit for DNA that is as low as 100 pM [158]. Additionally, Li et al. detected DNA samples using a paper-based isotachophoresis (ITP) device. This device consisted of concertina folding a paper strip with circular paper zones on each paper, and it was able to perform low-voltage ITP focusing of DNA. A fluorescence microscope with filter was used to measure the fluorescence of each fold of paper [198]. Recent advances in various detection methods in both medical and environmental applications include metal ions [199,200], fluoride ions [201], phenolic pollutants (Figure 8C) [202], antibiotic resistance genes (Figure 8D) [203], drug analysis of adrafinil [204], immunoassay [205], and visualization of cancer cells [206].

The issues with using paper as a substrate is that it also contains compounds that emit fluorescence signals under UV light, which creates a large background noise resulting in a more difficult sensing. Hence, to overcome these challenges, more sensitive and cost-effective fluorescent readers and direct coupling methods are being investigated for accurate measurements [207].

In summary, μPADs are moving in the direction of improved qualitative and quantitative detection capabilities. However, the main issue is still the sensitivity, robustness, and accuracy of the paper-based diagnostic devices compared to their conventional market competitors. Additionally, the long-term storage capability of the reagents should also be investigated for various detection protocols. With innovations in many of the biological and chemical detection techniques, simplified protocols would be suitable for adoption in paper-based platforms that would result in higher end quality products with lesser cost and complexity.

## 5. Conclusions and Future Perspectives

In this review, we discussed the various 2D and 3D fabrication techniques, flow control methods, and the applications of μPADs, which demonstrate numerous advantages such as low cost, fast reaction using low volume, simplicity, and biodegradability. The fabrication methods of µPADs were developed in parallel with dozens of microfabrication techniques and it has expanded into 3D techniques, which possess the complex problem-solving capability for multiple sequential reactions. Other advantages of 3D µPADs include increasing reaction speed and sequential delivery of reagents with free direction. The simple fabrication steps require stacking, folding, and a combination of 2D fabrication techniques. Furthermore, a variety of flow control methods were developed, permitting useful analytical platforms to be used in a wide range of applications related with health diagnostics, environmental monitoring, and drug analysis.

The future directions of various fabrication techniques will include commercially available mass production methods for convenient product development. In this field, we expect inkjet printing, flexographic printing, screen printing, and laser cutting at first to achieve rapid device fabrication and throughput as well as diversity in the use of various hydrophobic inks or chemicals for deposition on the μPADs. Additionally, the cost of fabrication will be reduced further with improvement in the resolution of patterning.

In terms of fluid handling, the flow control techniques, whether they are geometric, chemical or mechanical, need to be matured further for use in a variety of flow situations. The flow control systems at the moment either lack in sensitivity and reproducibility, or are complicated and expensive to produce.

In terms of commercialization and industrialization of µPADs, paper-fluidics is still in its transition stage. Although the recent growth of this field was at a tremendous pace, it still lacks a rapid and simple high-throughput manufacturing process without expensive chemicals or extra equipment to be successfully mass produced in the industry. Additionally, a full understanding of the fluid flow for both water and organic fluids on a variety of surfaces ranging from hydrophilic/hydrophobic to omniphilic and omniphobic should be well understood for producing an effective, high-performance device. Finally, the detection methods used must be low cost with higher sensitivity for achieving true POC diagnostics applications in resource-limited settings. Therefore, the focus should be on the development of µPADs with integrated flow control methods, which have advanced characterization with accuracy and precision of flow manipulation that is compatible with high-volume fabrication.

## Figures and Tables

**Table 1:** Summary of fabrication techniques for creating microfluidic paper-based analytical devices.

Fabrication Techniques	Equipment	Reagents	Advantages	Drawbacks	Ref.
Photolithography	Lithography equipment, mask aligner, hot plate	Positive or negative photoresist	High resolution	Expensive equipment and reagents, complex steps	[4,14,15,16,17]
Wax Printing	Wax printer, hot plate	Solid wax	Simple and fast fabrication process	Low resolution, requires a heating step	[18,19,20,21,22,23,24,25,26,27]
Plasma Treatment	Vacuum plasma reactor, masks, hot plate, microplasma generator device	AKD, fluorocarbon	Reduces the cost of materials such as AKD or fluorocarbon	High cost, requires masks depending on different designs	[28,29,30,31]
Plotting	Plotter	Hydrophobic ink (PDMS, wax), permanent marker, pen	Low cost, a physically flexible device	Low resolution, unstable liquid barrier	[19,32,33,34,35]
Inkjet Printing	Customized inkjet printer	Hydrophobic chemical, AKD, UV curable acrylate ink	High resolution, requires only a printer to fabricate µPAD	Requires customized inkjet printers	[37,38,39,40,41,42,43]
Laser Printing	Laser printer	Commercial toner	High resolution, simple to print using commercial device	Mostly requires additional heating step, limitation of materials	[44,45]
Flexographic Printing	Customized printing equipment	Polystyrene, PDMS	Applicable to roll-to-roll process, no requirement for heating step	High cost, requires complex preparation and cleaning, printing quality depends on surface roughness of paper	[46,47,48]
Stamping	PDMS or metallic stamp	Commercial ink	Low cost, easy to fabricate, ink storage capability, suitability for rapid prototyping in lab environment	Inconsistent results, low resolution, requires a preparation step	[49,50,51,52]
Chemical Vapor-phase Deposition	Deposition equipment	Hydrophobic chemicals such as poly(o-nitrobenzyl methacrylate), PPX, chlorosilane	Hydrophilic channels in paper are not affected by solvents, simple steps	High-cost instrumentation	[53,54,55,56]
Wet Etching	Mask	TMOS, NaOH	Simple, quick	Low resolution, requires a mask depending on the design	[57]
Hand-held Corona Treatment	Corona generator, PMMA mask, nitrogen gun	OTS, hexane, water, nitrogen	Quick, cost effective, simple	Hard to mass produce, requires washing step	[58]
Screen-printing	Mask for patterning	Wax, UV curable ink, carbon, silver/silver chloride	Low cost, simple fabrication steps	Low resolution, unadaptable to mass production	[59,60,61,62,63,64,65,66]
3D Printing	3D printer	PDMS, 3D printer resin	Fast and accessible to mass production	Resolution depends on 3D printer, expensive 3D printing machine	[67,68]
Spraying	Acrylic mask, UV/Vis light	Commercial water repellent product, scholar glue	Easy-to-use, equipment-free method	Low resolution and uniformity	[69,70]
Knife Plotter	Computer, plotter	None	Sharp boundary, simple, reduces the fabrication time, can be scaled up	Wastage of remaining paper, requires additional barrier or cover	[71,72]
Craft Cutting	Digital craft cutter	None or fluoroalkyltrichlorosilane	Lightweight, flexible, portable, disposable nature	Requires external pumping mechanism, low resolution	[73,74,75]
Embossing	Plastic molds, adhesive tape	Silane	Flexible and foldable devices	Low resolution, susceptible to contamination	[76]
Laser Cutting	Laser cutting machine	None	Rapid fabrication time, highly reproducible cutting, comparatively inexpensive laser cutting machine	Susceptible to contamination	[77,78,79,80]
